# Comparison of *msp* genotyping and a 24 SNP molecular assay for differentiating *Plasmodium falciparum* recrudescence from reinfection

**DOI:** 10.1186/s12936-019-2695-0

**Published:** 2019-03-18

**Authors:** Joseph Fulakeza, Sarah McNitt, Jimmy Vareta, Alex Saidi, Godfrey Mvula, Terrie Taylor, Don P. Mathanga, Dylan S. Small, Jacek Skarbinski, Julie R. Gutman, Karl Seydel

**Affiliations:** 1 Department of Biomedical Sciences, University of Malawi, College of Medicine, P/Bag 360, Chichiri, Blantyre 3, Malawi; 2 Department of Cell and Molecular Biology, Michigan State University, East Lansing, MI USA; 3 Blantyre Malaria Project, University of Malawi College of Medicine, Blantyre, Malawi; 4 Department of Osteopathic Medical Specialties, College of Osteopathic Medicine, Michigan State University, East Lansing, MI USA; 5 Department of Statistics, The Wharton School, University of Pennsylvania, Philadelphia, USA; 6 The Permanente Medical Group, Oakland, CA USA; 7 Malaria Branch, Division of Parasitic Diseases and Malaria, Center for Global Health, US Center for Disease Control and Prevention, Atlanta, USA

**Keywords:** Malaria, *Plasmodium falciparum*, *msp* genotyping, 24 SNP genotyping, Reinfection, Recrudescence

## Abstract

**Background:**

Current World Health Organization guidelines for conducting anti-malarial drug efficacy clinical trials recommend genotyping *Plasmodium falciparum* genes *msp1* and *msp2* to distinguish recrudescence from reinfection. A more recently developed potential alternative to this method is a molecular genotyping assay based on a panel of 24 single nucleotide polymorphism (SNP) markers.

**Methods:**

Performance parameters of these two genotyping methods were compared using data from two recently completed drug efficacy trials. Blood samples from two anti-malarial therapeutic trials were analysed by both *msp* genotyping and the 24 SNP assay. Additionally, to conserve time and resources, the statistical program R was used to select the most informative SNPs for a set of unrelated Malawian samples to develop a truncated SNP-based assay for the region surrounding Blantyre, Malawi. The ability of this truncated assay to distinguish reinfection from recrudescence when compared to the full 24 SNP assay was then analysed using data from the therapeutic trials.

**Results:**

A total of 360 samples were analysed; 66 for concordance of *msp* and SNP barcoding methodologies, and 294 for assessing the most informative of the 24 SNP markers. SNP genotyping performed comparably to *msp* genotyping, with only one case of disagreement among the 50 interpretable results, where the SNP assay identified the sample as reinfection and the *msp* typing as recrudescence. Furthermore, SNP typing was more robust; only 6% of samples were uninterpretable by SNP typing, compared to 19.7% when *msp* genotyping was used. For discriminating reinfection from recrudescence, a truncated 6 SNP assay was found to perform at 95.1% the accuracy of the full 24 SNP bar code.

**Conclusions:**

The use of SNP analysis has similar sensitivity to the standard *msp* genotyping in determining recrudescence from reinfection. Although more expensive, SNP typing is faster and less work intensive. Limiting the assay to those SNPs most informative in the geographical region of interest may further decrease the workload and the cost, making this technique a feasible and affordable alternative in drug efficacy trials.

**Electronic supplementary material:**

The online version of this article (10.1186/s12936-019-2695-0) contains supplementary material, which is available to authorized users.

## Background

In the face of continuing emergence of anti-malarial drug resistance, therapeutic trials of novel drugs, or combinations of known drugs, remain a critical part of a defensive strategy for combating this high-morbidity disease. A challenge with such studies arises in areas where malaria is endemic, such as sub-Saharan Africa, where individuals experience frequent infections. For example, the Chikhwawa district in southern Malawi has an estimated inoculation rate of 183 infective bites per person per year [1]. In such high transmission settings, it is imperative to determine whether infections occurring during the course of therapeutic drug trials are due to a recurrence of the initial infection (recrudescence) or a new infection (reinfection). This is a critical distinction, as recrudescence signifies resistance of the infection to the anti-malarial regimen, while a reinfection merely reflects a high transmission setting.

To distinguish between these two possibilities, each infection is characterized or “fingerprinted” using molecular genotyping of highly polymorphic alleles. Genotypes of the infection at enrollment, prior to the administration of the trial drug, are then compared with the post-treatment infection. Recrudescence is characterized by identical molecular fingerprints at both time points. In reinfection, on the other hand, the molecular fingerprints at the two-time points differ.

Genotyping to distinguish between reinfection and recrudescence is an effective approach because of the wide range of genetic heterogeneity among individual *Plasmodium falciparum* parasites, which are responsible for the majority of human malaria morbidity and mortality. Genetic diversity within this species is augmented by recombination events during its obligate sexual stage within the mosquito vector. Genetic diversity is particularly evident in high transmission settings like Malawi and other sub-Saharan African countries.

The current gold standard recommended by the World Health Organization (WHO) for distinguishing genetically distinct parasites involves genotyping the merozoite surface protein 1 and 2 (*msp1* and *msp2*) genes to identify length polymorphisms [2, 3]. *msp* genotyping is widely used and, because of the extensive polymorphism of *msp1* and *msp2* [4], is highly sensitive and specific. However, it is labour-intensive and time consuming, involving nested polymerase chain reaction (PCR) reactions and gel electrophoresis, and is therefore prone to amplification errors from contamination as well as subjective interpretation of band size on gel imaging.

An alternative method, a Taqman-based 24 single nucleotide polymorphism (SNP) barcode [5] has the potential to replace the cumbersome *msp1* and *msp2* genotyping [5]. The barcode method is less time and labour-intensive, as it uses only one round of DNA amplification and does not require electrophoresis. The results are relatively easy to interpret, and reactions can be monitored in real time. The particular 24 SNPs incorporated into this assay were chosen not only for their high minor allele frequency but also for their distribution across the 14 chromosomes of the *P. falciparum* genome, providing a high-resolution platform for distinguishing two closely related parasites [6]. Before the barcode method can be recommended as a replacement for genotyping *msp1* and *msp2*, the two techniques must be compared in terms of their capacity to accurately distinguish recrudescence from reinfection. In an effort to compare the performance characteristics of the two assays, results from both assays on samples collected in the course of two recent drug efficacy trials in Malawi were compared.

In addition to comparing *msp* genotyping to the originally designed 24 SNP barcode, an attempt was made to customize the barcode for Malawi. The original 24 SNP barcode was developed using sequences from parasites from West Africa and Southeast Asia. For drug efficacy studies in a smaller geographical area, a smaller number of SNPs might be sufficient to distinguish recrudescence from reinfection; this would increase the efficiency and decrease the costs of the assay.

In addition to its usefulness in distinguishing recrudescence from reinfection, the 24 SNP barcode is also often used to determine whether an infection represents a single genotypic clone or a mixture of multiple clones. Given that the malaria parasite is haploid during the blood stage in the human host, a single clone will reveal homozygous results at all 24 SNPs, whereas a mixed infection would reveal heterozygosity at one or more of the SNPs. Although identifying mixed infections was not the focus of this study, the truncated assay was also evaluated for this application as this is a common use of the 24 SNP assay.

## Methods

### Study area and population

To compare the barcode method versus *msp* genotyping, this study utilized 66 paired samples from cases of recurrent malaria infection during two therapeutic efficacy studies conducted among children age 6 to 59 months in Malawi between July 2011 and November 2012 at Machinga District Hospital and between March and July of 2014 at Machinga, Nkhotakota, and Karonga District Hospitals [7]. Consented, eligible children with fever or history of fever and *P. falciparum* mono-infections with 1000–200,000 asexual parasites/µl were enrolled (day 0) and followed on days 1, 2, 3, 7, 14, 21, 28, and 42 in the earlier study, and through day 28 in the later study. In addition, children were seen on any other day if they were ill. At each visit, blood was collected for thick and thin smears and for molecular testing on filter papers (Whatman 3MM). Patients with microscopically detected parasites on any day after day 3 were deemed to have recurrent parasitaemia and samples were tested by both *msp* and SNP genotyping methods as described below to differentiate recrudescence from reinfection. All genotyping was performed blinded to patient information.

For the evaluation of a geographically relevant truncated bar code, samples were obtained from patients (n = 294) admitted to the pediatric research ward at Queen Elizabeth Central Hospital in Blantyre, Malawi from January to June during the years 2009 and 2011. Peripheral blood was collected on FTA cards (Whatman 3MM) at admission and SNP genotyping was performed as described below.

### DNA extraction

DNA was extracted from blood spots using QIAamp DNA Mini and Blood Mini kit (Qiagen, Hilden, Germany) as per manufacturer’s instructions.

### *msp1* and *msp2* genotyping

A nested PCR was used to amplify the polymorphic repetitive regions block 2 of *msp1* [8] and block 3 of *msp2* [9] of all day 0 and day of recurrent parasitaemia samples [10]. Parasite DNA samples from standard laboratory strain 3D7 were included in each genotyping run as positive controls whilst water was included in each run as a negative control. The sequences of the primers and their respective positions in the respective genes are presented elsewhere [11]. In the first nested reaction, oligonucleotide primer pairs corresponding to conserved sequences spanning the polymorphic regions of the two genes were included.

Using a template from the product generated in the first reaction, five separate second nested reactions were then performed, using in each case specific primer pairs for MAD20, K1, and RO33 families of the *msp1* block 2, and the FC27 and 3D7/IC families of the *msp2* repeats as listed elsewhere [12], in order to determine the presence of allelic variants for *msp1* and *msp2*. Amplifications were performed on a BIO-RAD T100TM thermocycler (Hercules, CA) [12] and separated on a 2% gel for both *msp1* and *msp2*.

### Full 24 SNP and truncated SNP genotyping

Samples from all cases of recurrent parasite infection were genotyped using the molecular barcode assay as described previously [6]. The positions of the SNPs genotyped were as described in the original paper and annotated in PlasmoDB version 5.0. Parasite DNA samples from 3D7 were included in each genotyping run as positive controls and water was used as the negative control.

In brief, a master mix consisting of 2.95 µl of commercial grade nuclease-free water, 0.05 µl of the 40× Taqman SNP assay and 5 µl of the Taqman Universal PCR Master Mix (Applied Biosystems, Foster City, CA) per reaction was added to each well of the 96 well real-time PCR plate pre-loaded with 2 µl (10 ng) of each parasite DNA sample. Parasite DNA samples were then amplified on the Step One Plus real-time PCR instrument (Applied Biosystems, Foster City, CA, USA).

### Analysis

Samples were classified as either recrudescence, reinfection, or uninterpretable by each method, as follows. For *msp1* and *msp2* genotyping, the number and size of the electrophoresed bands on the day of recurrent parasitaemia and day 0 were compared. Bands less than 500 bp in size were considered the same if they were within 10 bp difference in size. If the number and size of bands representing allelic types on day 0 was equal to the number and size of bands observed on the day of recurrent parasitaemia, the recurrent episode of parasitaemia was considered a recrudescence (treatment failure). Otherwise, it was considered a new infection. Samples lacking a distinct band in any of the five amplification reactions were re-tested. Samples that twice failed to amplify were considered uninterpretable.

For SNP analysis, PCR amplification results of both the full 24 SNP barcode and truncated barcodes were completed using Applied Biosystem’s Proprietary Allelic Discrimination software version 2.2.2. Where the software did not give genotype calls directly, allele calls were made manually by examining both the amplification plot and the multi-component plot. A single allelic difference between day 0 and the day of recurrent infection was considered sufficient to call the infections different (i.e. reinfection) due to the high-resolution power of the allelic discrimination software used. Similarly, if a single allele was homozygous at day 0 but heterozygous at follow up, it was considered a new infection because the addition of a new allele represents a new parasite clone. Conversely, a heterozygous allele at day 0 changing to a homozygous allele at follow up could be due to the persistence of one strain of a mixed infection, with simultaneous clearance of the second strain and, therefore, in the absence of any other allelic differences, the case was designated as recrudescence and not reinfection.

For the SNP genotyping assays, if more than 2/3 of the SNPs (i.e. 17 of the full 24 SNP assay and 5 of the 6 SNPs of the truncated assay) were identical and the remaining SNPs failed to amplify, then the case was labelled recrudescent. Sensitivity analysis showed that variation of this cutoff by 3 SNPs in either direction resulted in the reclassification of only one case (a recrudescent sample being reclassified as uninterpretable). If 1/3 or more SNPs (8 for the full barcode and 2 for the truncated assay) failed to amplify for at least one of the time points of a matched sample, and there were no other SNP differences indicating a reinfection, then testing was repeated. If no additional SNPs were amplified the sample was labelled as uninterpretable.

Samples uninterpretable by either genotyping method were excluded from the final analysis. For the purposes of this study, *msp* genotyping was considered the gold standard, and results obtained from the barcode method were compared to the *msp* method using Stata version 10.1 (Stata, College Station, TX) to assess sensitivity and specificity of the barcode method.

The 24 SNP barcode was optimized for Malawi using the statistical package R (RStudio, Boston, MA). SNP calls were formatted into a 294 × 24 matrix where the 294 rows contained SNP calls from individual samples and the 24 columns represented SNPs 1–24. A function was created that would analyse and compare all pairs of rows (samples) using all possible combinations of 6, 8, 10, and 12 SNPs. The SNP combinations that yielded the maximum number of different rows (i.e. most often correctly identified a sample as being unique from all others) were subsequently labelled as truncated versions of the full 24 SNP assay optimized for our Malawian data set. The R functions specifically used are outlined in Additional File 1. The optimized truncated 6 SNP assay was then used to identify recrudescence and reinfection among the matched samples from the two therapeutic drug trials as described above, and results compared to both the full barcode and to *msp* genotyping.

## Results

**Fig. 1:**
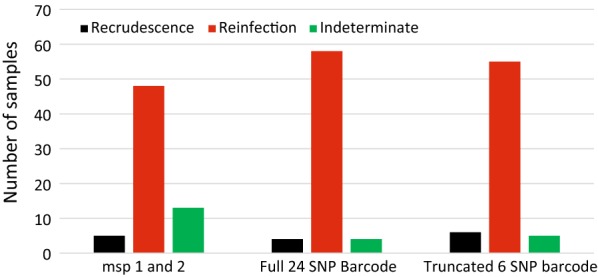
Genotyping results by *msp*, full 24 SNP, and truncated 6 SNP methods

Paired samples from 66 children were genotyped to distinguish between recrudescence and reinfection. Of these, *msp1* and *msp2* classified 5 (7.6%), 48 (72.7%), and 13 (19.7%) as recrudescence, reinfection, and uninterpretable, respectively (Fig. 1). Using the 24 SNP molecular barcode, 4 (6.1%), 58 (87.9%), and 4 (6.1%) were classified as recrudescence, reinfection, and uninterpretable, respectively, while 6 (9.1%), 55 (83.3%), and 5 (7.6%) were classified as recrudescence, reinfection and uninterpretable by the truncated 6 SNP barcode (Fig. 1).

When comparing the performance of the molecular barcode to the standard practice of *msp1* and *msp2* as the gold standard for detecting reinfection, sensitivity was 100% (95% CI 90.4–100%), specificity was 75.0% (95% CI 21.9–98.7%), positive predictive value was 97.9% (95% CI 87.3–99.9%), and negative predictive value was 100% (95% CI 31.0–100%) (Table 1).

**Table 1:** Performance of the barcode using *msp1* and *msp2* as the gold standard

	MSP		Total
	Reinfection	Recrudescence	
24 SNP barcode			
Reinfection	46	1	47
Recrudescence	0	3	3
Total	46	4	50

		Estimated value (%)	95% CI
Sensitivity		100	90.4–100
Specificity		75.0	21.9–98.7
Positive predictive value		97.9	87.3–99.9
Negative predictive value		100	31.0–100

The performance of *msp1* and *msp2* was also tested using the molecular barcode as the gold standard (Table 2). Sensitivity was 97.9% (95% CI 87.3–99.9%), specificity was 100% (95% CI 31.0–100%), positive predictive value was 100% (95% CI 90.4–100%), and negative predictive value was 75.0% (95% CI 21.9–98.7%). The overall correlation between the two methods was high with only one case showing a discrepancy (phi coefficient = + 0.86, p = 0.0002 via two-tailed Fisher Exact Probability Test).

**Table 2:** Performance of *msp*1 and *msp*-2 using the 24 SNP barcode as the gold standard

	24 SNP barcode		Total
	Reinfection	Recrudescence	
MSP			
Reinfection	46	0	46
Recrudescence	1	3	4
Total	47	3	50

	Estimated value (%)	95% CI
Sensitivity	97.9	82.5–98.4
Specificity	100	31.0–100
Positive predictive value	100	90.4–100
Negative predictive value	75.0	21.9–98.7

**Fig. 2:**
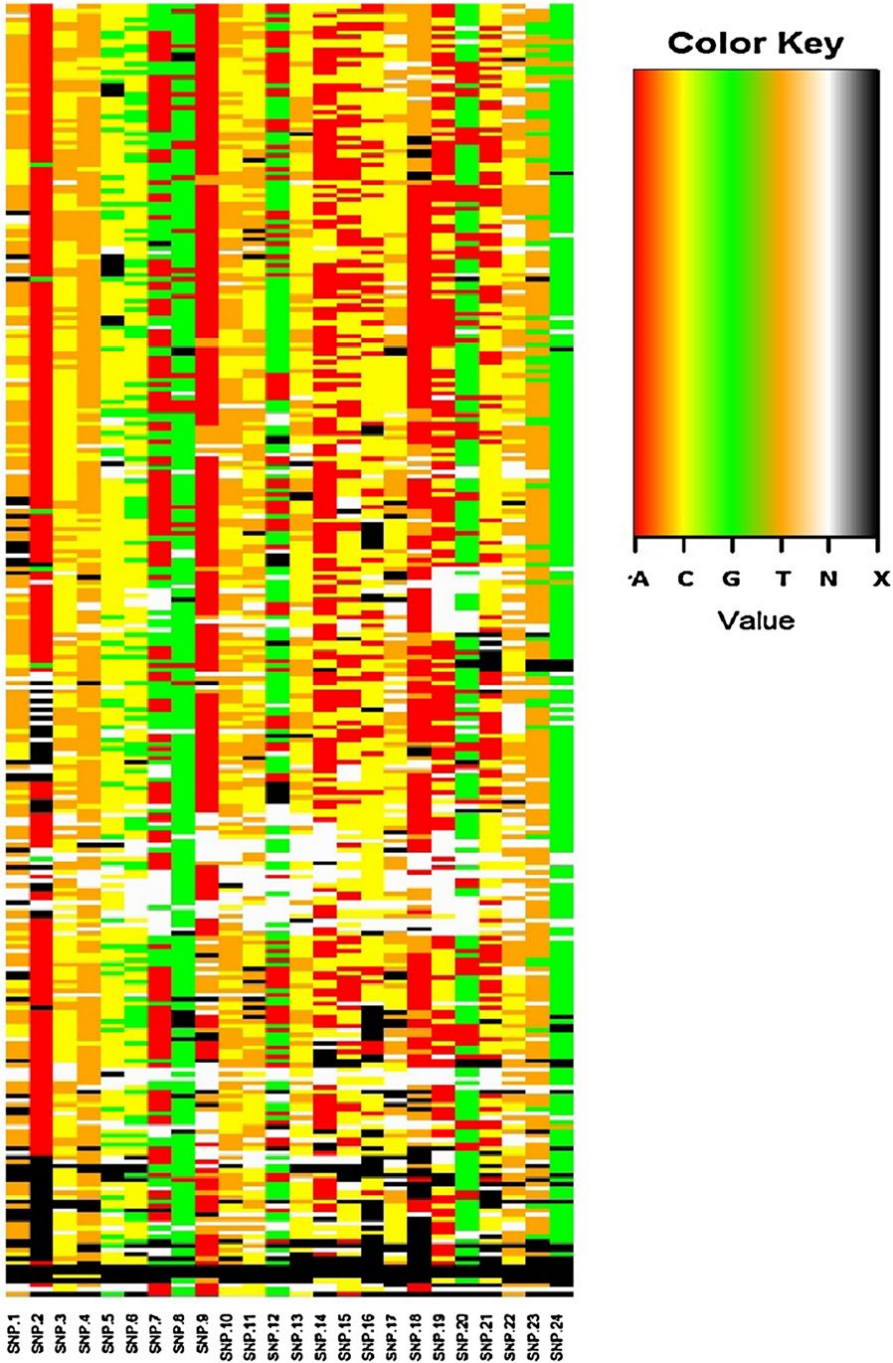
Heterogeneity of individual SNP’s among Malawian samples. In the key, values “A”, “C”, “G” and “T” indicate their respective nucleotide. Values labeled “N” represent a heterozygous or mixed finding at the respective SNP, while “X” represents missing values that failed to amplify during the PCR process

Some SNPs have extremely low variability in Malawian samples and therefore offer little in the way of genetic information (for instance, the vast majority of SNP 2 results are adenosine, whereas SNP 24 is almost always a guanosine) (Fig. 2). Limited heterogeneity at specific SNPs adds little additional information to the genetic “fingerprint” of a sample. This observation led to the effort to optimize the 24 SNP barcode for Malawi—excluding certain SNPs from the full panel without compromising the barcode’s ability to determine infection uniqueness.

The resulting optimized barcode of only 6 SNPs could, within the data set from Queen Elizabeth Central Hospital, distinguish two unique infections from each other over 98% of the time when compared to the full 24 SNP barcode. Similar test performances were found with a subset of 8, 10, and 12 SNPs (Table 3).

**Table 3:** Optimized subsets of the 24 SNP barcode (N = 294 samples)

	6 SNP barcode						8 SNP barcode		10 SNP barcode		12 SNP barcode	
SNP	3	7	11	15	19	22	4	14	9	10	5	17
Accuracy in calling uniqueness (%)	98.5						99.5		99.8		99.9	
Accuracy in determining heterozygosity (%)	78.0						79.7		83.6		89.3	
% amplification success	98	97	95	95	97	96	94	93	96	97	93	90
Minor allele frequency	0.31	0.32	0.44	0.50	0.43	0.49	0.16	0.29	0.19	0.40	0.21	0.40

In contrast to the ability to distinguish unique infections, the truncated bar code did not perform as robustly at identifying mixed infections. The 12 SNP barcode was only able to determine 89.3% of the mixed infections called by the 24 SNP bar code, suggesting that heterozygosity tends to occur at alleles less useful for showing the distinction between genotypes.

To determine how well these theoretically optimized barcodes could differentiate between two unique infections within the same individual (reinfection), they were then used to analyse the matched sample sets from the two therapeutic drug trials. The results were compared to both *msp* genotyping and the full 24 SNP barcode (Table 4).

**Table 4:** Comparison of the truncated barcodes to both the full 24 SNP assay and to *msp* genotyping

	6 SNP Barcode						8 SNP Barcode		10 SNP Barcode		12 SNP Barcode		24 SNP Barcode
SNP	3	7	11	15	19	22	4	14	9	10	5	17	Remaining
Accuracy in identifying reinfection using 24 SNP barcode as the gold standard	95.1						95.1		96.7		96.7		100%
Accuracy in identifying reinfection using *msp* genotyping as the gold standard	91.8%						91.8%		93.9%		93.9%		93.9%

For comparison of the full 24 SNP barcode and the truncated barcodes, a total of five uninterpretable samples from the original 66 samples were excluded. For comparison of the truncated barcode and *msp* genotyping, 17 samples were excluded because they were uninterpretable by *msp* (n = 13) or by the truncated barcode (n = 5), with one sample overlapping. The overall sensitivity for detecting reinfection using the 6 SNP barcode compared to the full 24 SNP assay was 94.8%, and specificity was 100%.

## Discussion

Molecular genotyping is a reliable method of distinguishing recrudescence from reinfection in anti-malarial drug efficacy trials. Although the WHO recommends *msp1* and *msp2* genotyping as a gold standard for distinguishing recrudescence from reinfection in areas with endemic malaria [3], this method is time consuming and labour-intensive [6]. The novel 24 SNP barcode method, which is considerably quicker and less labour intensive, performed comparably to *msp* genotyping for distinguishing recrudescence from reinfection (sensitivity 100% and specificity 75%).

The barcode method designed by Daniels et al. [6] uses 24 highly polymorphic SNPs that are broadly distributed across the *P. falciparum* genome and not in linkage disequilibrium, to clearly discriminate alleles in mixed genome samples. Daniels et al. demonstrated that no two parasites known to be of independent origin had the same allele signature. In addition, they showed that there was generally good agreement between the barcode and *msp1* and *msp2* genotyping with respect to identifying mixed parasite samples.

This study established that the two methods are comparable in their ability to distinguish recrudescence from reinfection. Both assays have advantages and disadvantages. The *msp* genotyping method resulted in more uninterpretable calls in our hands, with more reactions failing to generate distinct bands. Our levels of uninterpretable results were similar to those in other large scale studies [13, 14]. However, it is significantly less costly to use, with per sample reagent estimates of $3.60 for the *msp* method and $11.45 for the SNP-based assay. Although the ease of the SNP assay allows for higher throughput, when technician salaries as well as the cost of repeating uninterpretable results are included in the per assay estimate, the *msp* method is still cheaper with a cost of approximately $14.36 per sample as compared to $18.66 for the SNP based method. However, truncation of the SNP assay from 24 to 6 locations decreases the overall cost to $10.25 per sample, less than the *msp* method. Furthermore, the SNP assay requires more specialized equipment, with the requirement of a real-time PCR machine. In contrast, the *msp* based assay relies on the more universally available conventional PCR machines and standard gel electrophoresis. The SNP based assay has the advantage of being higher throughput—requiring only one amplification step, whereas two are required in the *msp* method. The SNP method also avoids the tedious step of loading samples onto an agarose gel.

In an effort to minimize time and resources while maintaining the equal discriminatory ability of the assay, the possibility of truncating the barcode was explored. Various studies have demonstrated surprisingly high levels of genetic relatedness among *P. falciparum* isolates from the same geographic region [15–18]. This suggests that, within a limited geographical region, the 24 SNP barcode might be truncated to eliminate any of the 24 SNPs which might be non-informative. The findings indicate that in areas of northern Malawi the truncated 12 SNP barcode suggested above determines whether a new infection is unique from the last cleared infection (i.e. reinfection) 96.7% of the time when compared to the full 24 SNP barcode. This would decrease time and resources by almost half. Further truncation to a set of the six most informative SNPs would still result in 95.1% accuracy in determining recrudescence from reinfection. The truncated bar code does not, however, perform as robustly when used for determining single from mixed infections, likely due to the increased advantage of detecting low frequency heterogeneity at more sites in the full bar code assay.

A significantly higher proportion of samples were uninterpretable by *msp* genotyping than by SNP barcode. Uninterpretable results seen in the *msp1* and *2* based assays were due to poor amplification or inability to determine amplicon size on gel electrophoresis, while uninterpretable results in the SNP based assay were due to poor amplification. Of the 13 samples that were uninterpretable by *msp* genotyping and 4 by the full barcode, only one sample was uninterpretable by both methods, suggesting assay specific failures rather than general poor quality of DNA. In the analysis of the truncated barcode, this led to a large apparent drop in the accuracy of the 6 SNP barcode in determining reinfection when using *msp* genotyping rather than the full 24 SNP barcode as the gold standard—from 95.1 to 91.8%. Using the full 24 SNP panel as the gold standard, the 6 SNP barcode only missed three cases of reinfection within the total 61 samples analysed in that comparison. Using the *msp* genotyping as the gold standard, even though there was only one additional sample disagreement, the drop in total sample number from 61 to 49 led to a 45/49 agreement, i.e. a 91.8% accuracy.

In comparing Tables 3 and 4, it is notable that the truncated barcodes did not perform as well in the analysis using *msp* results as the gold standard. For instance, the 6 SNP assay’s accuracy in discriminating unique infections dropped from 98.5 to 95.1%. One possible explanation may be that the 6 SNP optimized barcode was proposed based on a large data set of unique genotypes from a geographic area immediately surrounding Blantyre. This SNP set was then used to analyse samples from Machinga, Nkhotakota, and Karonga district hospitals, which are 120, 375, and 825 km away from Blantyre, respectively. Geographic genotype variance has been shown to be very focal [15–18]. While it was originally assumed that a barcode optimized using samples from southern Malawi would still perform satisfactorily in analysing samples from northern Malawi, perhaps a different truncated SNP panel, optimized using a large database generated from unique infections in northern rather than southern Malawi, would perform more accurately for these samples collected at some distance from Blantyre. The R algorithm could be used to determine optimum SNPs for other geographically distinct malaria endemic regions as well.

The major limitation of this study is the small sample size. This limitation is magnified by the fact that there are very few recrudescent infections by either measurement method. This leads to an exceedingly low number of recrudescent infections to be compared by the two methods. An additional limitation is the inability to amplify all alleles in all samples—in both the *msp* and the SNP based methods. Although this non-amplification was not biased towards any specific location, the cutoff of allowing up to seven non-amplified alleles could result in the misclassification of reinfections as recrudescence. Although this limitation would be equally present with either technique, it is of increased concern in the *msp* assay where there was a high percent of non-amplification (19%). Finally, the choice of samples from geographically separated regions for design and validation of the truncated bar code may have resulted in less than ideal performance of the truncated barcode.

## Conclusions

This study showed comparable results from *msp1* and *msp2* genotyping and the SNP-based barcodes. The major downfall of the SNP-based method—increased cost—could be effectively ameliorated with the design of a truncated set of SNPs. In Malawi, a subset of 6 SNPs performed almost as well as the originally designed 24 SNP method, thus substantially decreasing cost without compromising data quality.

## Additional file

**Additional file 1.** R code for generating truncated bar code.
